# Protective Role of Chrysin against Mercury Chloride-Induced Testicular Damage via Oxidative Stress, Inflammation, Apoptosis, Endoplasmic Reticulum Stress, and Autophagy Pathways

**DOI:** 10.1007/s12011-026-04993-y

**Published:** 2026-01-20

**Authors:** Serpil Aygörmez, Mustafa Makav, Ebru Karadağ Sarı, Elif Dalkılınç, Hamit Uslu, Şaban Maraşlı

**Affiliations:** 1 Department of Biochemistry, Faculty of Veterinary Medicine, Kafkas University, Kars, Türkiye; 2 Department of Physiology, Faculty of Veterinary Medicine, Kafkas University, Kars, Türkiye; 3 Department of Histology-Embryology, Faculty of Veterinary Medicine, Kafkas University, Kars, Türkiye; 4 Department of Biochemistry, Faculty of Veterinary Medicine, Atatürk University, Erzurum, Türkiye; 5 Department of Physiology, Faculty of Medicine, Erzincan Binali Yıldırım University, Erzincan, Türkiye

**Keywords:** Mercury chloride, Chrysin, Endoplasmic reticulum stress, Apoptosis, Autophagy

## Abstract

The aim of this research was to examine the potential ameliorative effects of chrysin (CHR) against mercuric chloride (HgCl_2_)-induced testicular damage in rats. For this purpose, rats were divided into four groups: Control, CHR, HgCl_2_ and HgCl_2_ + CHR. HgCl_2_ was administered intraperitoneally at a dose of 1.23 mg/kg, and CHR was administered orally at a dose of 50 mg/kg for 7 days. Biochemical, molecular and immunohistochemical analyses were performed to determine the effect of treatment-mediated changes in the testicular tissue. Based on the results obtained in testicular tissue, administration of HgCl_2_ was observed to lower antioxidant markers, elevate malondialdehyde (MDA) levels, and increase inflammatory marker expression in rat testicular tissue. It also led to reduced testosterone levels. Additionally, there was a decrease in the expression of antiapoptotic B-cell lymphoma 2 (Bcl-2) an apoptosis marker while the levels of Caspase-3 and Bcl-2-associated X protein (Bax) were found to be higher. The endoplasmic reticulum stress marker protein kinase R-like ER kinase (PERK) and the autophagy marker Beclin-1 showed strong immunoreactivity. Additionally, HgCl_2_ + CHR treatment were found to significantly reduce oxidative stress, inflammation, apoptosis, endoplasmic reticulum stress and autophagy processes in testicular tissue. In conclusion, HgCl_2_ administration to rats caused testicular tissue damage compared to the other groups, but CHR treatment alleviated this damage. Overall, this demonstrates the potential ameliorative mechanisms of CHR as a possible agent for HgCl_2_-induced testicular damage.

## Introduction

Heavy metals are prevalent environmental contaminants due to their persistence and resistance to biodegradation, making their removal from ecosystems challenging [1]. Exposure to mercury, the most toxic heavy metal, through food, soil, air and water poses a threat to health [2]. The most toxic form is mercury chloride (HgCl_2_) because it easily forms organo-mercury complexes with proteins [3–5]. In addition, this crystalline and odorless compound can be easily absorbed as a result of its high solubility [6]. Exposure to HgCl_2_ has genotoxic, hematotoxic, neurotoxic and reproductive toxic effects [5, 7]. It triggers oxidative stress by disrupting the antioxidant-prooxidant balance and increasing reactive oxygen species [2, 3]. Oxidative stress, on the other hand, causes pathological disorders in the reproductive system as a result of increased reactive oxygen species damaging macromolecules [4, 8]. The possible mechanism of testicular toxicity following HgCl_2_ exposure is still unknown. However, changes such as oxidative stress, apoptosis, and inflammation have been reported to may play a role [7]. HgCl_2_ reacts with cellular thiols, weakening antioxidant defense systems. Furthermore, HgCl_2_ is a potent apoptosis inducer through an increase in nuclear factor kappa-B (NF-κB) levels [3]. HgCl_2_ causes deformation in seminiferous tubules and Leydig cells and testicular toxicity, leading to adverse effects on the reproductive system and reducing reproductive performance [9]. The testicles are essential for male fertility as they produce testosterone and support spermatogenesis. HgCl_2_ induces testicular damage by generating reactive oxygen species and activating specific signaling pathways [10]. It has been reported that the use of plant-derived compounds with high antioxidant content can prevent diseases or treat damage caused by oxidative stress in order to protect the antioxidant-prooxidant balance that is disrupted as a result of oxidative stress [11].

Flavonoids, a group of plant-derived polyphenolic compounds, have widespread beneficial effects on human health. Flavonoids are abundant in bee products, vegetables, and fruits [12]. These compounds are rich in biological activity due to their hydroxyl content [13]. Chrysin (5,7-dihydroxyflavone (CHR)), found in plants such as *Oroxylum indicum*,* Lactarius deliciosus* and *Radix Scutellaria*, is a natural flavonoid [14]. It has become the focus of many researchers due to its antiapoptotic, antiinflammatory and antioxidant properties [15, 16]. Due to the keto and hydroxyl groups in its structure, it is known to have biological effects such as antioxidant and antiinflammatory properties. It also has a direct pharmacological effect on fertility and reproduction [17, 18]. It has been reported that its effect on male fertility is due to strengthening the antioxidant defense on the testicle by neutralizing free radicals [14, 19]. Studies have shown that CHR inhibits the production of reactive oxygen species and the release of proinflammatory cytokines such as tumor necrosis factor-α (TNF-α) in testicular tissue. It reduces lipid peroxidation and improves glutathione (GSH) levels and antioxidant activities. It has also been reported to improve levels of apoptotic markers such as antiapoptotic B-cell lymphoma 2 (Bcl-2), Bcl-2-associated X protein (Bax) and Caspase-3. Thanks to these effects, CHR has been reported to exhibit antioxidant, antiinflammatory, and antiapoptotic properties in testicular tissue [13, 20–22].

Since there have been few studies on testicular damage caused by HgCl_2_ exposure, this study aimed to examine how CHR affects HgCl_2_-induced testicular damage. The research used biochemical, molecular, and immunohistochemical analyses to assess organ damage.

## Materials and methods

### Chemicals

HgCl_2_ (purity ≥ 99.0%, EC-No: 231-299-8, Tekkim, Bursa, Türkiye), CHR (purity 97%, CAS-No: 480-40-0, Sigma-Aldrich, St. Louis, MO, USA), phosphate buffered saline (PCode: 1003268877, Sigma-Aldrich, St. Louis, MO, USA), xanthine (purity ≥ 99%, CAS-No: 69-89-6, Sigma-Aldrich, St. Louis, MO, USA), bovine serum albumin (purity ≥ 96%, CAS-No: 9048-46-8, Sigma-Aldrich, St. Louis, MO, USA), 1,1,3,3-tetraethoxypropane (purity ≥ 96%, CAS-No: 122-31-6, Sigma-Aldrich, St. Louis, MO, USA), glutathione (purity ≥ 98.0%, CAS-No: 70-18-8, Sigma-Aldrich, St. Louis, MO, USA), cumene hydroperoxide (purity 80%, CAS-No: 80-15-9, Sigma-Aldrich, St. Louis, MO, USA), 5,5’-dithiobis(2-nitrobenzoic acid) (purity 99%, CAS-No: 69-78-3, Sigma-Aldrich, St. Louis, MO, USA), nitro blue tetrazolium (purity 99%, CAS-No: 298-83-9, Sigma-Aldrich, St. Louis, MO, USA), xanthine oxidase (CAS-No: 9002-17-9, Sigma-Aldrich, St. Louis, MO, USA), hydrogen peroxide (purity 30%, Sigma-Aldrich, USA), perchloric acid (purity 70.0–72.0%, CAS-No: 7601-90-3, Sigma-Aldrich, St. Louis, MO, USA), tris(hydroxymethy)amino-methane (CAS-No: 77-86-1, Sigma-Aldrich, St. Louis, MO, USA), ammonium sulfate (CAS-No: 7783-20-2, Sigma-Aldrich, Burlington, MA, USA), 2-thiobarbituric acid (CAS-No: 504-17-6, Sigma-Aldrich, Burlington, MA, USA), methanol (CAS-No: 67-56-1, Sigma-Aldrich, Burlington, MA, USA), trichloroacetic acid (CAS-No: 76-03-9, Sigma-Aldrich, Burlington, MA, USA), sodium carbonate (CAS-No: 497-19-8, Sigma-Aldrich, Burlington, MA, USA), copper(II)chloride (purity 97%, CAS-No: 7447-39-4, Sigma-Aldrich, St. Louis, MO, USA), folin-ciocalteu’s phenol (Sigma-Aldrich, Burlington, MA, USA), potassium chloride (CAS-No: 7447-40-7, Sigma-Aldrich, Burlington, MA, USA), disodium ethylenediaminetetraacetate dihydrate (CAS-No: 6381-92-6, Sigma-Aldrich, Burlington, MA, USA), hydrochloric acid (purity 37%, CAS-No: 7647-01-0, Sigma-Aldrich, Burlington, MA, USA), potassium tartrate (CAS-No: 6381-59-5, Sigma-Aldrich, Burlington, MA, USA), sodium hydroxide (CAS-No: 1310-73-2, Sigma-Aldrich, Burlington, MA, USA), copper sulfate (CAS-No: 7758-98-7, Sigma-Aldrich, Burlington, MA, USA) were used.

### Rat Characteristics and Ethics Committee Approval

Total of 28 male *Wistar Albino* rats, weighing 220–250 g, were used in this study. The animals were housed in cages in a controlled room with a constant temperature of 24–25 °C and a 12-hour light-dark cycle. They had access to unlimited water and standard food. Ethical approval for the study was obtained from the Kafkas University Animal Experiments Local Ethics Committee (KAU-HADYEK) (Approval number: KAU-HADYEK/2025 − 219).

### Experimental Groups

In this study, 4 groups were formed with 7 animals in each group. The groups were;

- **I-Control:** Saline was administered intraperitoneally for 7 days.
- **II-CHRA**: 50 mg/kg CHR was administered via oral gavage for 7 days [20].
- **III-HgCl**_**2**_: Rats were administered intraperitoneal 1.23 mg/kg HgCl_2_ in physiological saline for 7 days [3].
- **IV-**_**2**_** + CHR**: Rats were given 1.23 mg/kg HgCl2 intraperitoneally in physiological saline and 50 mg/kg CHR via oral gavage for 7 days.

At the end of the applications, all experimental animals were sacrificed under anesthesia with ketamine (35–50 mg/kg) and xylazine (5–10 mg/kg) intramuscularly in accordance with ethical rules. Testicular tissues were then removed, and one was stored at -80 °C for biochemical and molecular analyses. The other testicular tissue was preserved in 10% neutral buffered formaldehyde for histopathological examination.

### Oxidative Stress Analyses

Testicular tissue was diluted with 1.15% potassium chloride homogenized. To determine oxidative stress, malondialdehyde (MDA) and GSH levels, and glutathione peroxidase (GPx), catalase (CAT), and superoxide dismutase (SOD) activities were analyzed. The MDA level in testicular tissue was measured using the method reported by Placer et al. [23]. A pink color was formed as a result of the reaction between thiobarbituric acid (TBA) and MDA. The absorbance value of the pink color was measured at 532 nm using a NanoDrop (Bio-Tek, Gen5, Epoch, USA) device. Testicular tissue GSH level was determined using the method reported by Sedlak and Lindsay [24]. According to this analytical method, the yellow color is formed as a result of the reduction of 5,5-Dithiobis(2-nitrobenzoic) acid (DTNB) by sulfhydryl group compounds. The absorbance values ​​of this color were determined by spectrophotometric measurement at 412 nm (Bio-Tek, Gen5, Epoch, USA). Testicular tissue SOD activity measurement was performed according to the method reported by Sun et al. [25]. SOD activity measured at a wavelength of 560 nm (Bio-Tek, Gen5, Epoch, USA) is based on the reduction of nitrobluetetrazolium (NBT) by superoxide radicals produced by the xanthine-xanthine oxidase system. When the enzyme is insufficient, maximum reduction occurs, and a dark blue color is observed. If sufficient SOD is present, the enzyme converts superoxide anion to hydrogen peroxide (H_2_O_2_), reducing NBT reduction and causing a color change. Formazan formation is inversely proportional to enzyme concentration. The method reported by Aebi [26] was used for CAT measurement. CAT measurement principle is based on the ability of H_2_O_2_ to absorb light at 240 nm (Bio-Tek, Gen5, Epoch, USA). GPx activity was analyzed using the method developed by Lawrence and Burk [27]. The absorbance of the resulting color was measured at a wavelength of 412 nm (Bio-Tek, Gen5, Epoch, USA). The protein content of testicular tissue was determined by the method reported by Lowry et al. [28]. The protein content relies on complexing with peptide bonds using alkaline copper tartrate reagent. When phenol reagent is added to the copper-treated mixture, a purple-blue color formation is observed. This resulting color was measured at 650 nm (Bio-Tek, Gen5, Epoch, USA).

### Enzyme-Linked Immunosorbent Assay (ELISA) Analysis

Testosterone levels were analyzed by enzyme-linked immunosorbent assay **(**ELISA). Analysis was performed using a commercial ELISA kit (Cat. No: 201-11-5126) (Sunred, China) according to the manufacturer’s procedure. In this analysis, according to the manufacturer’s instructions, no sample or antibody labeled with Biotin and Streptavidin-HRP was added to the blank well; only Chromogen A, B, and stop solution were added. 50 µL of standard and 50 µL of Streptavidin-HRP were added to the standard wells. 40 µL of sample was added to the sample wells, followed by 10 µL of antibody and 50 µL of Streptavidin-HRP. It was then covered with a membrane and incubated for 60 min at 37 °C with gentle shaking. The membrane was then carefully removed and the plate wells washed. After the plate dried, 50 µL of chromogen solution A was added to each well, followed by 50 µL of chromogen solution B. The plate was mixed gently and incubated at 37 °C away from light for 10 min. Then 50 µL of stop solution was added to each well to stop the reaction (the blue color immediately turned yellow). After adding the stop solution, the optical density at 450 nm was measured using a spectrophotometer (Bio-Tek, Gen5, Epoch, USA).

### RNA Extraction and Real-Time Polymerase Chain Reaction (RT-PCR) Analysis

According to the manufacturer’s guidelines, total RNA was extracted from testicular tissue using QIAzol Lysis Reagent (Qiagen, Germany). The concentration and purity of the RNA samples were evaluated using a NanoDrop^®^ spectrophotometer (BioTek Epoch). cDNA was synthesized from 2 µg of total RNA using the High-Capacity cDNA Kit (Applied Biosystems, Thermo Fisher Scientific, Lithuania). RT-PCR was conducted using the Power SYBR Green Master Mix PCR kit (Qiagen, Germany) on the Rotor-Gene Q 5plex HRM platform (Qiagen, Germany). The mRNA levels of NF-κB, TNF-α, interleukin 1β (IL-1β), Bcl-2, Bax, Caspase-3, activating transcription factor 6 (ATF-6), and glucose-regulated protein 78 (GRP78) in the testicular tissues were analyzed in triplicate using gene-specific primers (Table 1). β-actin was used as reference gene. The relative gene expression levels were determined from the Ct value and calculated using the 2^−ΔΔCt^ method [29].

**Table 1:** Primer sequences of genes analyzed in RT-PCR

Gene	Accession number	Primers	Product Size (bp)
β-actin	NM_031144.3	**F**: GGAGATTACTGCCCTGGCTCCTAGC **R**: GGCCGGACTCATCGTACTCCTGCTT	155
NF-κB	NM_001415012.1	**F**: CCACTGTCAACAGCAGATGG **R**: TTTGCAGGCCCCACATAGTT	179
TNF-α	NM_012675.3	**F**: ATGGGCTCCCTCTCATCAGT **R**: GCTTGGTGGTTTGCTACGAC	106
IL-1β	NM_031512.2	**F**: GACTTCACCATGGAACCCGT **R**: GGAGACTGCCCATTCTCGAC	104
Bax	NM_017059.2	**F**: CCAGGACGCATCCACCAAGAAGC **R**: TGCCACACGGAAGAAGACCTCTCG	136
Bcl-2	NM_016993.2	**F**: TATATGGCCCCAGCATGCGA **R**: GGGCAGGTTTGTCGACCTCA	136
Caspase-3	NM_012922.3	**F**: GGAGCTTGGAACGCGAAGAA **R**: ACACAAGCCCATTTCAGGGT	169
ATF-6	NM_001107196.1	**F**: CACAGACACGGATGATGTCCAGTT **R**: GTCTGAGCAGAAGTGGCTGCT	136
GRP78	NM_013083.2	**F**: CTGATTCCGAGGAACACTGTGGTG **R**: CTTTTGTCAGGGGTCGTTCACCT	118

### Histopathological Examination

Testicular tissue samples taken from rats were fixed in a 10% formalin solution. Tissue Sect. 5 micrometers (µm) thick were taken from paraffin blocks prepared after routine histological procedures. Hematoxylin & Eosin (H&E) staining technique was used to show the general structure of the tissue.

### Immunohistochemical Analyses

Testicular tissues obtained from rats were fixed in 10% formaldehyde solution to determine the immunoreactivity of protein kinase R-like ER kinase (PERK) and Beclin-1, and subjected to the streptavidin-biotin peroxidase method. Phosphate-buffered saline (PBS, 0.1 M, pH 7.2) was used for all washing steps. Sections were incubated in 0.1 M PBS with 3% H₂O₂ for 15 min at room temperature, then boiled in citrate buffer solution (microwave oven, 800 W, 10 min). Blocking solution A (Invitrogen Histostain Plus Broad Spectrum (AEC)) was added to prevent non-specific binding. Then, PERK (Santa Cruz, sc-377400, diluted 1/100), Beclin-1 (Santa Cruz, sc-48341, diluted 1/100), and the primary antibody were applied to the sections and incubated overnight at + 4 °C. After the primary antibody, Broad Spectrum Antibody (Invitrogen Histostain Plus Broad Spectrum (AEC)) antibody, which is against the species for which the primary antibody was produced, was added to the sections and kept at room temperature for 15 min. After washing with PBS, HRP streptavidin (Invitrogen Histostain Plus Broad Spectrum (AEC)) was incubated at room temperature for 15 min. For chromogen application, DAB (3.3′-Diaminobenzidine) Substrate Solution was added, immunoreactivity was observed, and then modified Gill III hematoxylin was used as counterstaining. To confirm specific immunoreactivity, sections incubated with PBS instead of the primary antibody were passed through all steps. Two independent observers assessed the immunostaining intensity and density of the target cells using a semi-quantitative scoring system. The evaluation was done by assigning values ​​from 0 to 3 according to the characteristics of no staining (-), weak staining (+), moderate staining (++), and strong staining (+++). All prepared sections were evaluated and photographed using a light microscope (Olympus BX51, Olympus Optical Co. Ltd., Osaka, Japan).

### Statistical Analysis

The normality of the parameter values in each group was assessed using the Shapiro-Wilk test. One-way analysis of variance (ANOVA) was used to assess statistical differences and significance levels, while the Tukey test was used to determine group differences using GraphPad 10.3.1 (GraphPad Software, San Diego, CA, USA). The results that were presented as mean ± standard deviation (SD). The data obtained as a result of immunohistochemical examinations (taking the staining degree of cells showing immunoreactivity as a criterion) were analyzed using the SPSS 20.0 software. One-way analysis of variance (ANOVA) was performed to determine the differences between groups and the Tamhane test was used to compare the differences between groups. Statistical significance was determined at *p* < 0.05.

## Results

### Protective Effect of CHR on HgCl_2_-Induced Oxidative Stress in Testicular Tissue

**Fig. 1:**
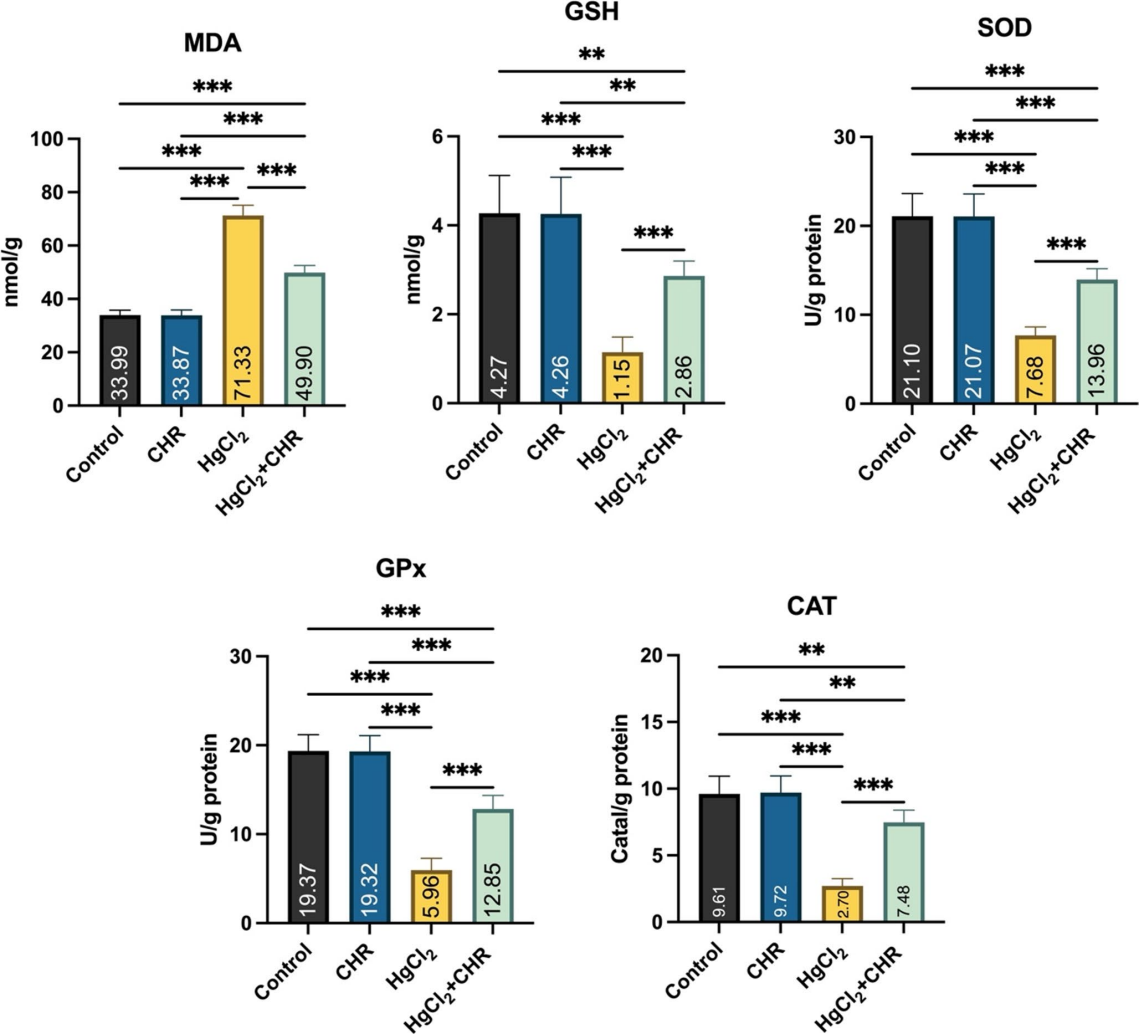
Effect of HgCl_2_ and CHR administration on oxidative stress parameters in testicular tissue. Values ​​for each group are given as mean ± standard deviation (SD). *p < 0.05, **p < 0.01, ***p < 0.001. (HgCl_2_: Mercury chloride, CHR: Chrysin, MDA: Malondialdehyde, GSH: Glutathione, SOD: Superoxide dismutase, GPx: Glutathione peroxidase, CAT: Catalase)

Oxidative stress markers MDA and GSH levels, and SOD, GPx, and CAT activities are shown in Fig. 1. In the HgCl_2_ group, a statistically significant increase in MDA levels was observed compared to the control and CHR groups (*p* < 0.001). In the HgCl_2_ group, compared to the control and CHR groups, a decrease was detected in the levels of antioxidant markers GSH (*p* < 0.001) and SOD (*p* < 0.001), GPx (*p* < 0.001), and CAT (*p* < 0.001) activities. HgCl_2_ + CHR treatment reduced the MDA level, which was increased by HgCl_2_ administration (*p* < 0.001). HgCl_2_ + CHR treatment increased SOD, GPx, and CAT activities, while reducing GSH levels with HgCl_2_ administration (*p* < 0.001). There was no statistically significant difference between the control and CHR groups (*p* > 0.05).

### Protective Effect of CHR on HgCl_2_-Induced Testosterone Level in Testicular Tissue

**Fig. 2:**
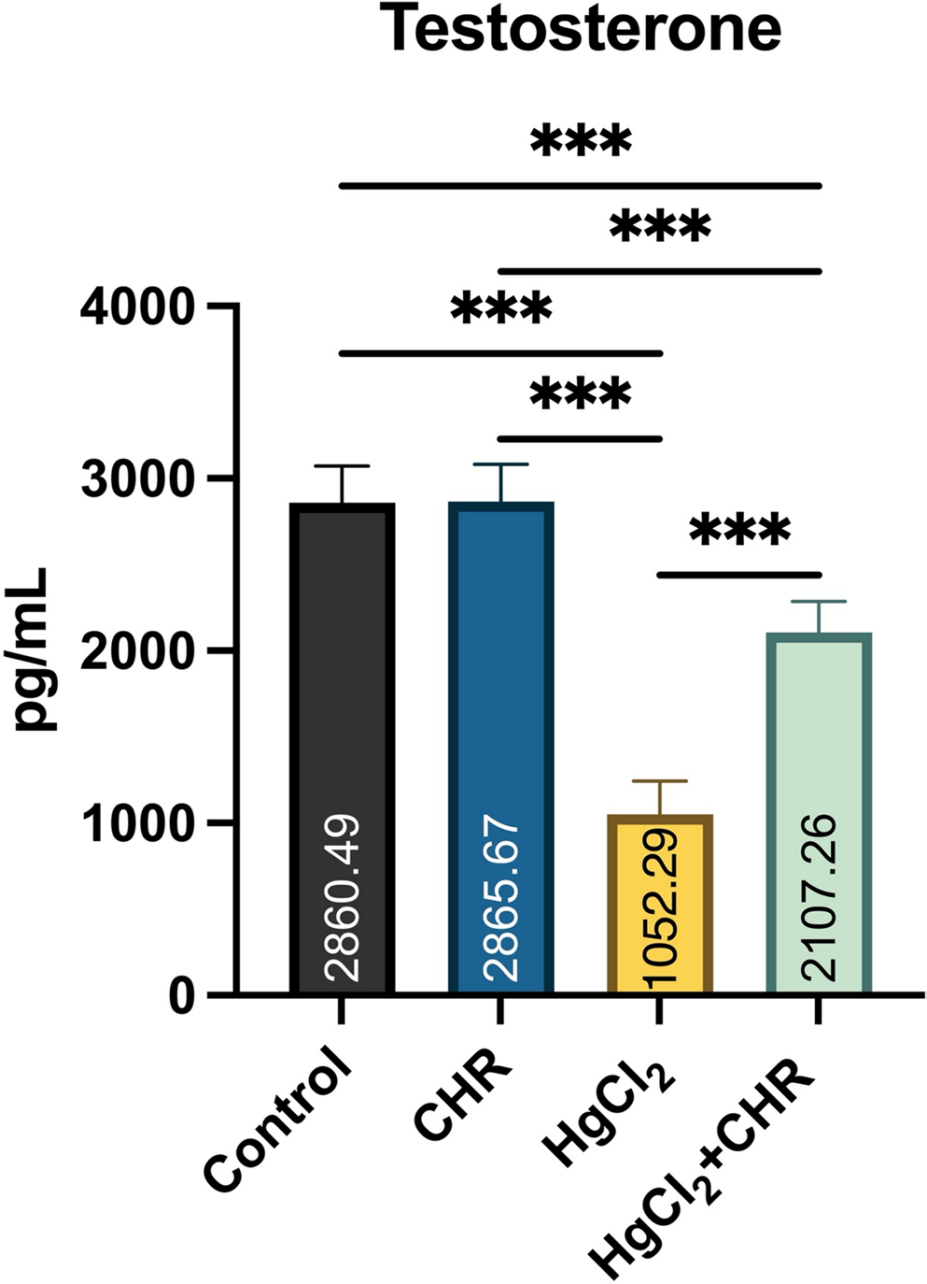
Effect of HgCl_2_ and CHR administration on testosterone levels in testicular tissue. Values ​​for each group are given as mean ± standard deviation (SD). *p < 0.05, **p < 0.01, ***p < 0.001. (HgCl_2_: Mercury chloride, CHR: Chrysin)

Testosterone levels in rats are shown in Fig. 2. In rats, HgCl_2_ administration resulted in decreased testosterone levels compared to the control and CHR groups (*p* < 0.001). HgCl_2_ + CHR treatment was found to increase testosterone levels, which were reduced by HgCl_2_ administration (*p* < 0.001). There was no statistically significant difference between the control and CHR groups (*p* > 0.05).

### Protective Effect of CHR on HgCl_2_-Induced Inflammation in Testicular Tissue

**Fig. 3:**
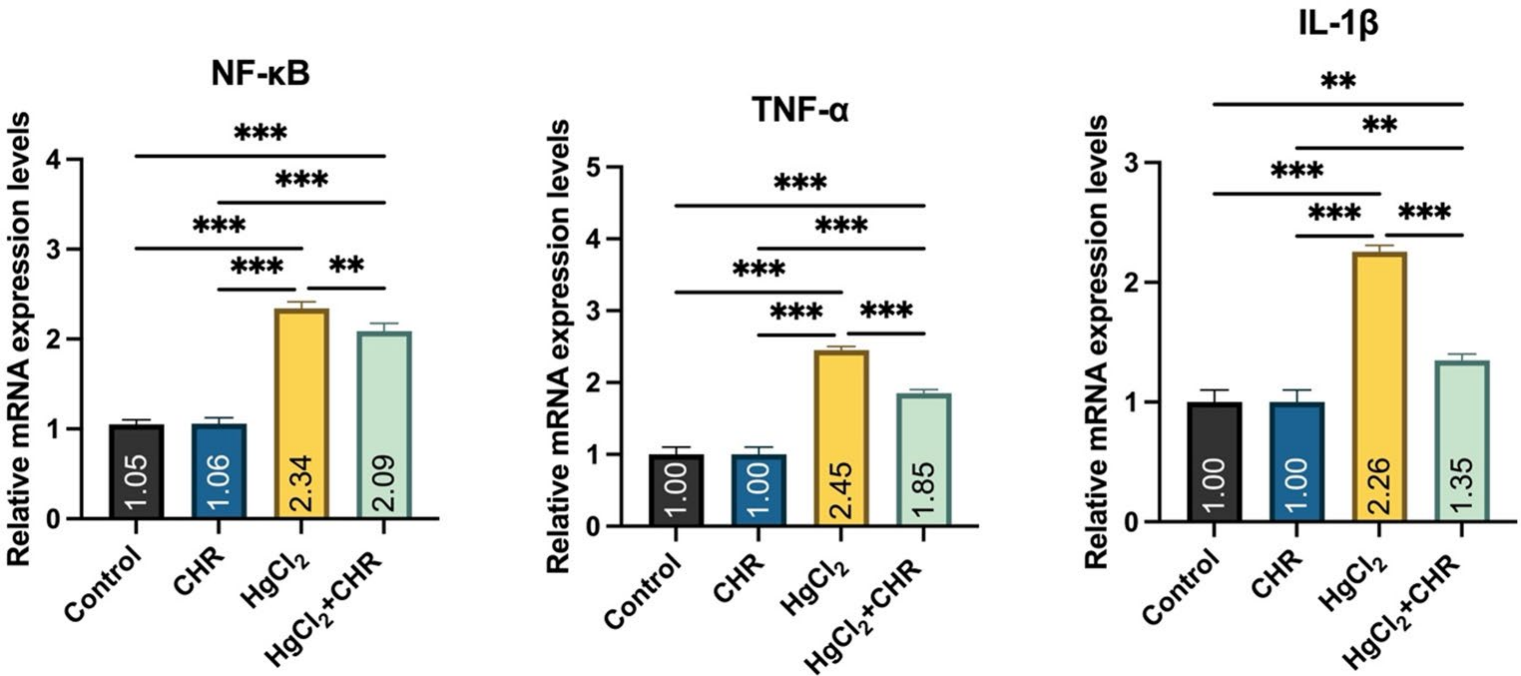
Effect of HgCl_2_ and CHR administration on inflammation parameters in testicular tissue. Values ​​for each group are given as mean ± standard deviation (SD). *p < 0.05, **p < 0.01, ***p < 0.001. (HgCl_2_: Mercury chloride, CHR: Chrysin, NF-κB: Nuclear factor kappa B, TNF-α: Tumor necrosis factor-α, IL-1β: Interleukin 1β)

mRNA expression levels of inflammation markers NF-κB, TNF-α, and IL-1β are presented in Fig. 3. In the HgCl_2_ group, NF-κB, TNF-α, and IL-1β mRNA expression levels were found to be increased compared to the control and CHR groups (*p* < 0.001). HgCl_2_ + CHR treatment was found to decrease NF-κB (*p* < 0.01), TNF-α (*p* < 0.001), and IL-1β (*p* < 0.001) mRNA expression levels, which were increased by HgCl_2_ administration. There was no statistically significant difference between the control and CHR groups (*p* > 0.05).

### Protective Effect of CHR on HgCl_2_-Induced Apoptosis in Testicular Tissue

**Fig. 4:**
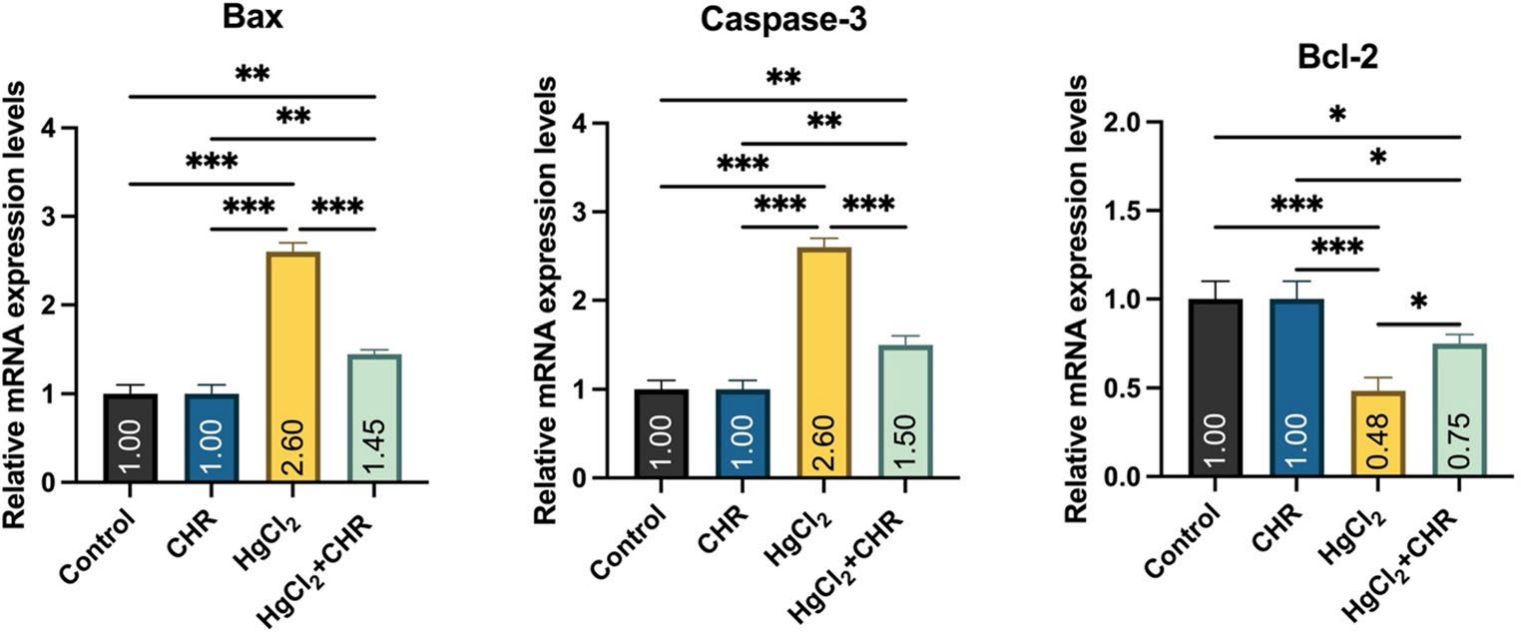
Effect of HgCl_2_ and CHR administration on apoptosis parameters in testicular tissue. Values ​​for each group are given as mean ± standard deviation (SD). *p < 0.05, **p < 0.01, ***p < 0.001. (HgCl_2_: Mercury chloride, CHR: Chrysin, Bcl-2: Antiapoptotic B-cell lymphoma 2, Bax: Bcl-2-associated X protein)

The RT-PCR results of the apoptosis parameters Bax, Bcl-2, and Caspase-3 are shown in Fig. 4. In the HgCl_2_ group, a significant increase in pro-apoptotic markers (Caspase-3, Bax) and a decrease in Bcl-2 were observed compared to the control and CHR groups (*p* < 0.001). HgCl_2_ + CHR treatment was found to significantly improve Bax and Caspase-3 levels, which were increased by HgCl_2_ administration (*p* < 0.001). Furthermore, HgCl_2_ + CHR treatment increased the Bcl-2 mRNA expression level, which had decreased with HgCl_2_ administration (*p* < 0.05). There was no statistically significant difference between the control and CHR groups (*p* > 0.05).

### Protective Effect of CHR on HgCl_2_-Induced Endoplasmic Reticulum Stress in Testicular Tissue

**Fig. 5:**
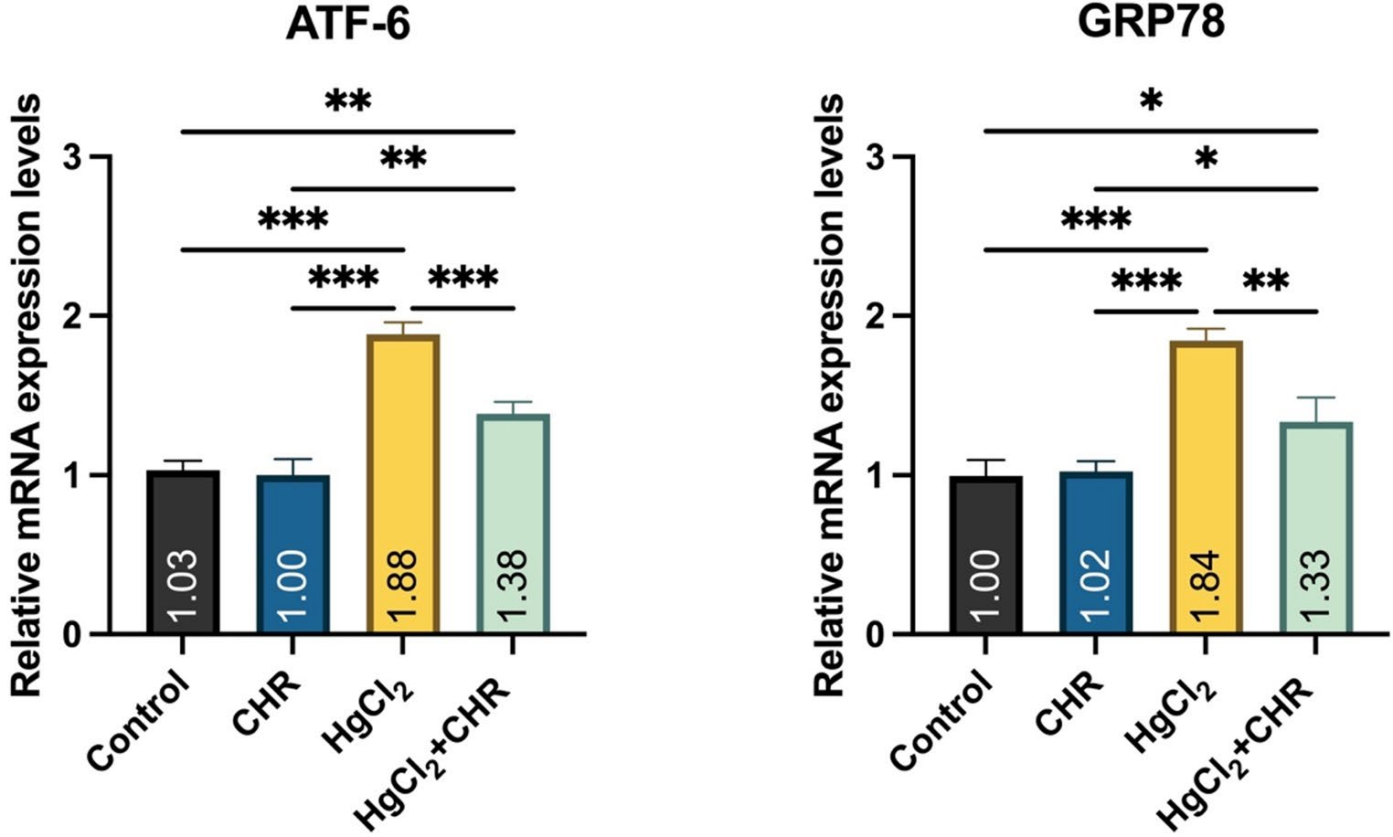
Effect of HgCl_2_ and CHR administration on endoplasmic reticulum stress parameters in testicular tissue. Values ​​for each group are given as mean ± standard deviation (SD). *p < 0.05, **p < 0.01, ***p < 0.001. (HgCl_2_: Mercury chloride, CHR: Chrysin, ATF-6: Activating transcription factor 6, GRP78: Glucose-regulated protein 78)

mRNA expression levels of endoplasmic reticulum stress markers ATF-6 and GRP78 are presented in Fig. 5. In the HgCl_2_ group, ATF-6 and GRP78 mRNA expression levels were found to be increased compared to the control and CHR groups (*p* < 0.001). HgCl_2_ + CHR treatment was found to reduce the increased ATF-6 (*p* < 0.001) and GRP78 (*p* < 0.01) mRNA expression levels observed with HgCl_2_ administration. There was no statistically significant difference between the control and CHR groups (*p* > 0.05).

### Histopathological Results

**Fig. 6:**
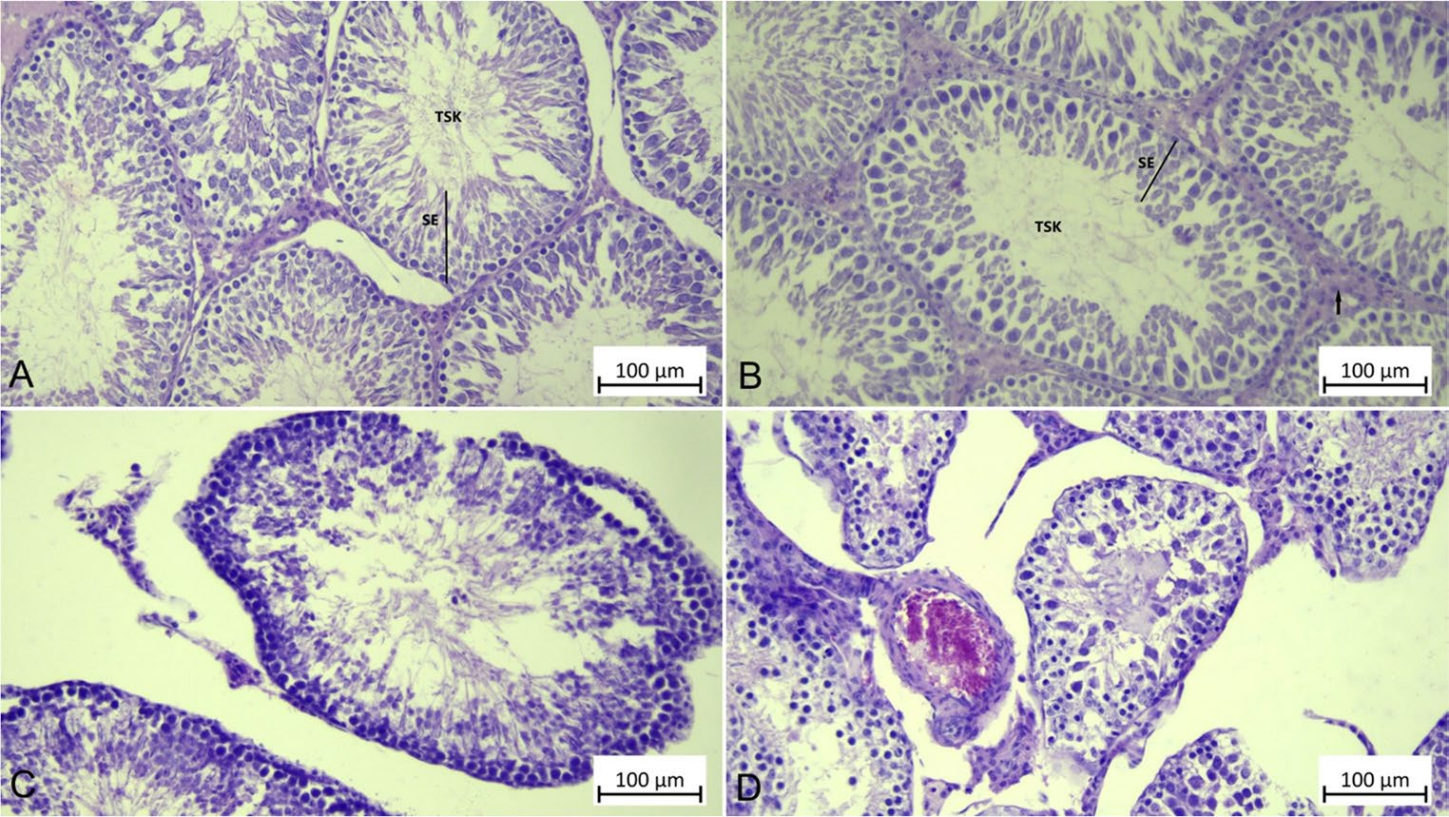
Effect of HgCl_2_ and CHR administration on H&E analysis in testicular tissue. H&E analysis results are indicated by TSK, SE, and arrow. (**A**) Control, (**B**) CHR, (**C**) HgCl_2_, (**D**) HgCl_2_ + CHR. (HgCl_2_: Mercury chloride, CHR: Chrysin, H&E: Hematoxylin & Eosin, TSK: Tubulus seminiferus contortus, SE: Seminiferous epithelium, arrow: interstitial space)

Control and CHR groups showed normal histological structure in the testicular tissues of the rats (Fig. 6A and B). In HgCl_2_ group rat testicular tissue examinations, irregularly shaped and deformed seminiferous tubules and interstitial space dilations were observed. Atrophic formations were observed in some areas within the seminiferous tubules, spermatogenic cells were shed into the lumen, and Leydig cells were detached in the interstitial space (Fig. 6C). Examinations of rat testicular tissue in the HgCl_2_ + CHR group revealed that the seminiferous tubules were of normal structure, with vacuolation and atrophy observed in some tubular structures, but significantly less so compared to rat testicular tissue in the HgCl_2_ group (Fig. 6D).

### Protective Effect of CHR on HgCl_2_-induced PERK and Beclin-1 Expression in Testicular Tissue

**Fig. 7:**
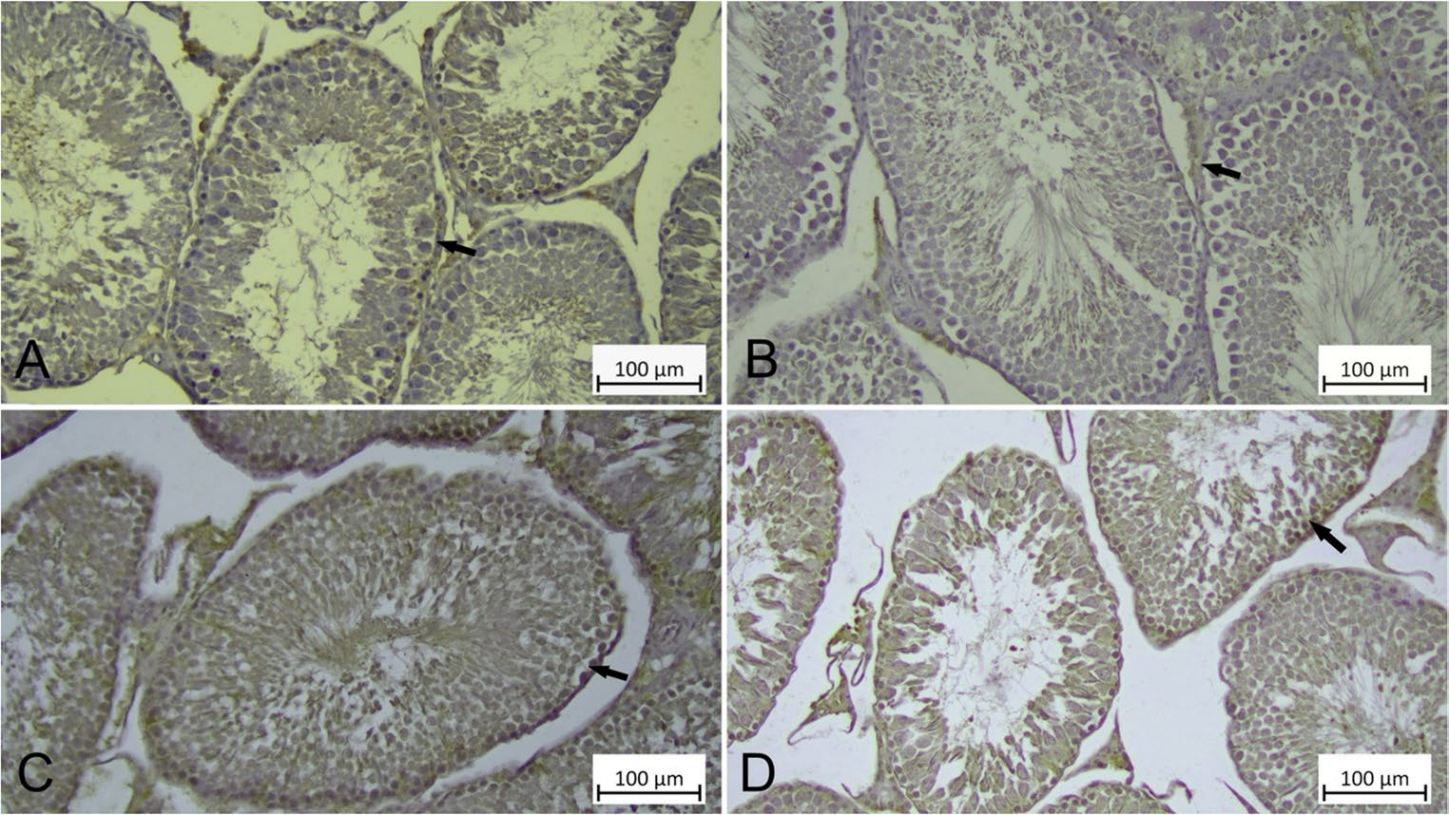
Effect of HgCl_2_ and CHR administration on PERK immunoreactivity in testicular tissue. Immunoreactivity for PERK is indicated by an arrow. (**A**) Control, (**B**) CHR, (**C**) HgCl_2_ (**D**) HgCl_2_ + CHR, PERK immunoreactivity. IHC staining. (HgCl_2_: Mercury chloride, CHR: Chrysin, PERK: Protein kinase R-like ER kinase)

**Fig. 8:**
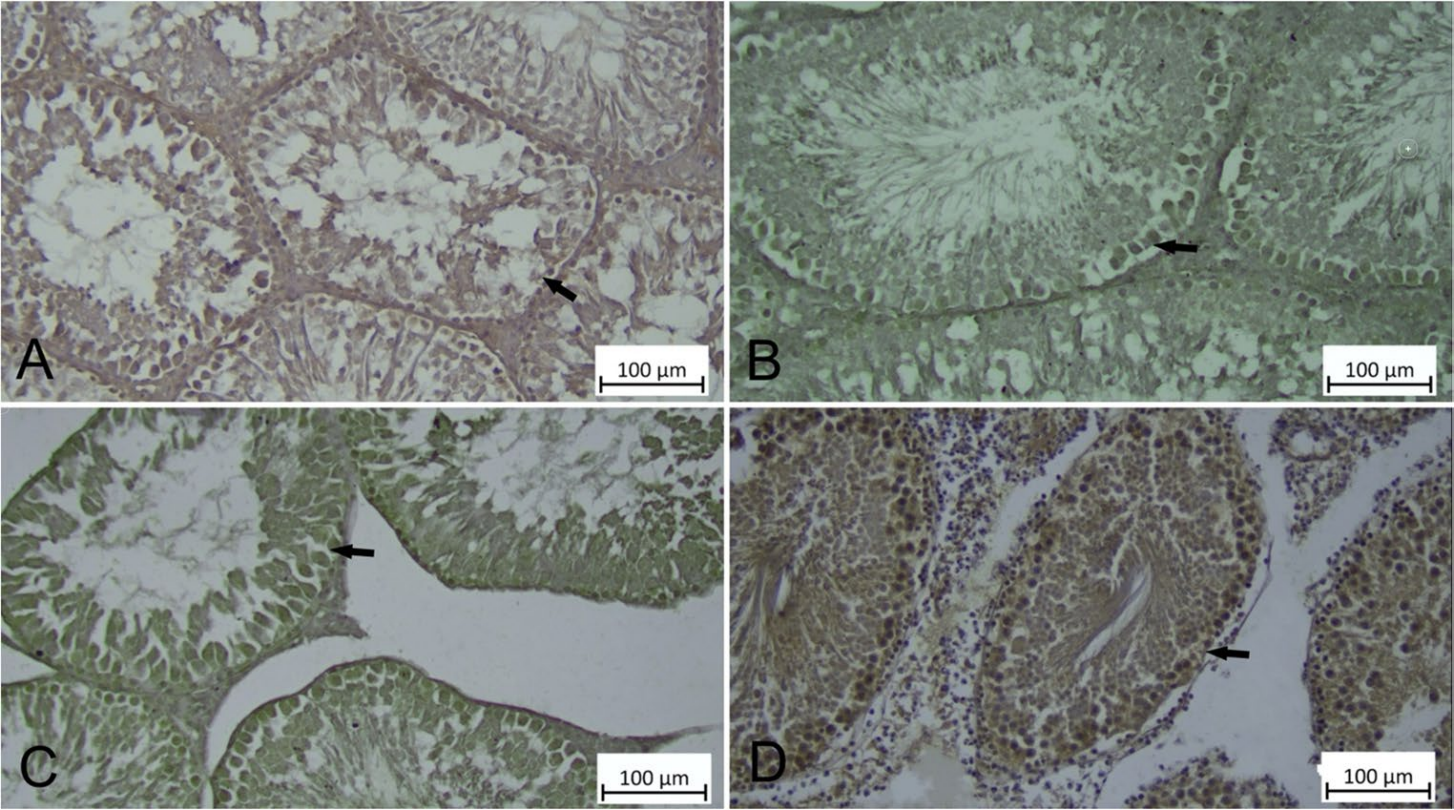
Effect of HgCl_2_ and CHR administration on Beclin-1 immunoreactivity in testicular tissue. Immunoreactivity for Beclin-1 is indicated by an arrow. (**A**) Control, (**B**) CHR, (**C**) HgCl_2_, (**D**) HgCl_2_ + CHR, Beclin-1 immunoreactivity, IHC staining. (HgCl_2_: Mercury chloride, CHR: Chrysin)

When PERK and Beclin-1 immunoreactivity was examined in all groups, it was determined that there was generally cytoplasmic PERK and Beclin-1 immunoreactivity in primary spermatocytes, spermatids and Leydig cells in all Control, CHR, HgCl_2_ and HgCl_2_ + CHR groups (Figs. 7 and 8). When the difference in immunoreactivity between the groups was examined, PERK and Beclin-1 immunoreactivity was detected at a weak level in the testicular tissue of rats in the Control (Figs. 7A and 8A), CHR (Figs. 7B and 8B) groups, at a strong level in the testicular tissue of rats in the HgCl_2_ group (Figs. 7C and 8C), and at a moderate level in the primary spermatocytes, spermatids, and Leydig cells in the testicular tissue of rats in the HgCl_2_ + CHR (Figs. 7D and 8D) group. As a result of the statistical analysis performed to compare PERK and Beclin-1 immunoreactivity values ​​according to the groups, it was determined that significantly stronger immunoreactivity was observed in primary spermatocytes, spermatids and Leydig cells in the HgCl_2_ group compared to all groups. In addition, it was determined that the HgCl_2_ + CHR group had moderate PERK and Beclin-1 immunoreactivity in primary spermatocytes, spermatids and Leydig cells compared to the Control and CHR groups (*p* < 0.001) (Table 2).

**Table 2:** Semiquantitative analysis of PERK and Beclin-1 immunoreactivity in testicular tissue

		Control	CHR	HgCl_2_	HgCl_2_ + CHR	F	Sig.
PERK	**Primary Spermatocytes**	0,8875 ± 0,5096^c^	0,7875 ± 0,4952^c^	2,6750 ± 0,5006^a^	1,7750 ± 0,6329^b^	53,856	< 0.001
	**Spermatids**	0,8750 ± 0,5287^c^	0,7875 ± 0,4952^c^	2,6750 ± 0,5006^a^	1,7750 ± 0,6329^b^	53,312	< 0.001
	**Leydig Cell**	0,9875 ± 0,2361^c^	0,9500 ± 0,2236^c^	2,7500 ± 0,3344^a^	1,8875 ± 0,5762^b^	106,496	< 0.001
Beclin-1	**Primary Spermatocytes**	0,9250 ± 0,4143^c^	0,8625 ± 0,3845^c^	2,7000 ± 0,3498^a^	1,8625 ± 0,5875^b^	77,165	< 0.001
	**Spermatids**	0,9875 ± 0,2361^c^	1,0250 ± 0,2913^c^	2,7875 ± 0,2841^a^	1,8875 ± 0,4173^b^	146,825	< 0.001
	**Leydig Cell**	0,9875±,2361^c^	1,0625 ± 0,2416^c^	2,8375 ± 0,2841^a^	1,8125 ± 0,3429^b^	189,280	< 0.001

^*^Different letters in each row indicate statistical differences

## Discussion

HgCl_2_, the salt of the heavy metal mercury, is known to cause various tissue damage. This tissue damage is associated with the activation of damage pathways caused by oxidative stress. For this reason, in this study, the effects of CHR, a natural flavonoid, on HgCl_2_-induced testicular damage were investigated biochemically, molecularly and immunohistochemically.

Testosterone, a steroid hormone in the testis, is synthesized and secreted by Leydig cells. Testosterone plays an important role in germ cell differentiation, spermatogenesis, and the development of the male reproductive system [30]. Heavy metals can change hormones due to their toxic effects on Leydig cells and, as a result, affect spermatogenesis and reduce testosterone levels [31–33]. In the presented study, it was found that the heavy metal HgCl_2_ reduced the testosterone level and the natural compound CHR treatment increased the decreased testosterone level. Studies have reported that heavy metals reduce testosterone levels [34, 35], while natural compounds increase testosterone levels in HgCl_2_ damage [10, 36].

Heavy metals disrupt the oxidant/antioxidant balance, causing oxidative stress [1, 37]. Heavy metals interact with amino groups, thiol and hydroxyl groups in proteins, forming protein complexes and disrupting the structures of enzymes by inhibiting their activity [38]. Reactive oxygen species that cause oxidative stress disrupt cell structure and cause tissue function loss [39, 40]. If the reactive oxygen species formed are not removed, they trigger cell death [37, 41]. Antioxidant enzymes constitute the defense line against oxidative stress in the body [42]. The resulting oxidative stress prevents the development of sperm cells in the testicular tissue and causes apoptosis [1, 37]. The presence of heavy metals in testicular tissue increases reactive oxygen species, which in turn impairs antioxidant activity [1, 43]. Oxidative stress, particularly when it leads to tissue damage due to impaired blood flow, is a critical factor in the pathogenesis of testicular dysfunction [44]. At the cellular level, it disrupts the delicate balance between the production of reactive oxygen species and the effectiveness of antioxidant defense mechanisms [44, 45]. This disrupted balance leads to oxidative damage in various cellular components, including proteins, lipids, and deoxyribose nucleic acid, ultimately contributing to testicular damage [44]. In the study, it was found that MDA levels increased, GSH levels and CAT, GPx and SOD enzyme activities decreased in the damage caused by HgCl_2_ in the testicular tissue of rats. As a result of these results, it was determined that reactive oxygen species increased and the increased reactive oxygen species created oxidative stress, and the resulting oxidative stress caused damage to the testicular tissue. In studies conducted on the subject, it has been reported that HgCl_2_ increases lipid peroxidation in the testicular tissue and triggers oxidative stress as a result of the depletion of naturally occurring antioxidants in the organism, and the resulting oxidative stress causes damage to the testicular tissue [7, 9]. In the presented study, we found that CHR, a natural flavonoid, reduces the increased MDA level and has a healing effect against oxidative stress by regulating the antioxidant balance. Studies have shown that CHR protects testicular tissue integrity in heavy metal toxicity by protecting proteins, DNA damage, sperm membranes, reducing lipid peroxidation and repairing cell damage by increasing enzymes [13, 15].

Inflammation is the body’s line of defense to eliminate harmful substances such as damaged cells, irritants, and pathogens [46]. For cellular functions, NF-κB regulates cellular activities such as inflammation, stress response and cell death [47, 48]. Inflammation begins with the onset of oxidative stress in the testicular tissue [49]. Activation of NF-κB suppresses steroidogenesis in Leydig cells by inhibiting steroidogenic enzyme transcription [50]. Inflammation resulting from increased reactive oxygen species causing oxidative stress affects male fertility [50, 51]. In the presented study, it was found that NF-κB, TNF-α and IL-1β mRNA expression levels increased with HgCl_2_ administration to rats, and this increase is thought to be caused by oxidative stress in the testicular tissue. Studies on the subject have reported that HgCl_2_ can trigger inflammatory activation due to oxidative stress caused by decreased antioxidant defense system and increased lipid peroxidation in testicular tissue, and the resulting inflammation can lead to tissue damage [52, 53]. In our study, it was determined that HgCl_2_ + CHR treatment improved inflammation in the testicular tissue by reducing the increased NF-κB, TNF-α and IL-1β mRNA expression levels. Studies emphasize that natural compounds have protective properties in testicular tissue due to their antioxidant and antiinflammatory properties against heavy metal damage due to the interaction between oxidative stress and inflammation [54, 55].

Increased reactive oxygen species in the testes leads to deterioration in the antioxidant defense system and fertility [37, 56]. Oxidative stress caused by increased reactive oxygen species and the increase in proinflammatory cytokines resulting from oxidative stress mediate apoptosis [51, 57]. Apoptosis is a programmed death with significant deoxyribose nucleic acid damage [58–60]. After the disruption of the mitochondrial membrane, Caspase-3 is activated and the apoptotic cascade begins [51]. Antiapoptotic Bcl-2 and proapoptotic Bax, which play a role in apoptosis, undergo conformational changes [57]. Apoptosis plays an important role in reproductive physiology in heavy metal damage [60]. This is highlighted by the increase in Caspase-3 and Bax levels and the decrease in Bcl-2 levels in testicular tissue during heavy metal damage [51, 58]. In the presented study, it was found that HgCl_2_ administration increased the expression levels of Bax and Caspase-3 mRNA, decreased the expression level of Bcl-2 mRNA, and as a result led to apoptosis in the testicular tissue of rats. Studies have shown that HgCl_2_ causes lipid peroxidation by increasing hydrogen peroxide production and reactive oxygen species in the cytoplasm of testicular tissue, resulting in damage to membranes and apoptosis [1, 61]. In our study, it was determined that HgCl_2_ + CHR administration prevented apoptosis by reducing Bax and Caspase-3 mRNA expression levels and increasing Bcl-2 mRNA expression levels in testicular tissue due to the antiapoptotic properties of CHR. In studies on the subject, it has been reported that CHR protects the testicular tissue from heavy metal damage by reducing oxidative stress due to its antioxidant properties and then regulating apoptotic genes [15, 62].

The number of unfolded proteins in the endoplasmic reticulum, which is responsible for the processing and synthesis of proteins, increases when oxidative stress occurs [63, 64]. Homeostatic imbalance in the endoplasmic reticulum causes endoplasmic reticulum stress [64]. The most typical marker of endoplasmic reticulum stress is GRP78. In Leydig cells, GRP78 increases with the progression of apoptosis [65]. Endoplasmic reticulum stress transducer transmembrane proteins first activate PERK and then ATF-6 [64, 66]. PERK and ATF-6 bind to GRP78, an endoplasmic reticulum chaperone [67]. In the presented study, it was determined that HgCl_2_ administration increased the mRNA expression levels of GRP78 and ATF-6 in the testicular tissue of rats in RT-PCR results, and there was strong PERK immunoreactivity in primary spermatocytes, spermatids and Leydig cells in the testicular tissue of rats in immunohistochemical results. Studies have stated that there is a relationship between oxidative stress, apoptosis and endoplasmic reticulum stress and that this relationship may have a negative effect on reproduction [60, 68]. In testicular tissue, HgCl_2_ + CHR treatment were found to reduce GRP78 and ATF-6 mRNA expression levels in RT-PCR results, while immunohistochemical results of PERK showed moderate PERK immunoreactivity in primary spermatocytes, spermatids and Leydig cells in testicular tissue of rats. In studies on the subject, it has been reported that CHR improves oxidative stress by reducing increased reactive oxygen species against different toxic substances and reduces endoplasmic reticulum stress after the improved oxidative stress regulates apoptotic genes [12, 67].

Autophagy, which is related to pathological and physiological processes such as cell metabolism, death and survival, is the adaptive mechanism of cells and tissues in the event of oxidative stress [58, 69]. It is also important for cellular homeostasis, removing damaged organelles and misfolded proteins from cells [70]. Bcl-2 combines with Beclin-1 to inhibit autophagy under physiological conditions [71]. Heavy metals trigger cell damage by causing autophagic death [58, 70]. Decreased Bcl-2 levels increase Beclin-1 levels [71]. In the presented study, the immunohistochemical result of HgCl_2_ administration in the testicular tissue of rats found that Beclin-1 had strong immunoreactivity in primary spermatocytes, spermatids and Leydig cells. It has been reported that heavy metals cause autophagy by increasing the level of Beclin-1 in testicular tissue as a result of inducing reactive oxygen species and apoptosis [60, 71]. In this study, the immunohistochemical result of HgCl_2_ + CHR treatment in the testicular tissue of rats revealed moderate immunoreactivity for Beclin-1 in primary spermatocytes, spermatids and Leydig cells. In a study, it was stated that CHR reduces apoptosis due to its antiapoptosis properties against different toxic substances in testicular tissue and improves the resulting autophagy [13].

## Conclusion

The results of the present study demonstrated that oxidative stress, inflammation, apoptosis, autophagy, and endoplasmic reticulum stress are closely related to HgCl_2_-induced testicular damage, and CHR has protective effects against these HgCl_2_-induced damage. The protective effects of CHR are likely due to reducing oxidative stress, inflammation, apoptosis, autophagy, and endoplasmic reticulum stress. Overall, the potential healing mechanisms of CHR as a possible agent in HgCl_2_-induced testicular damage have been revealed.

### Study Limitations

It should be noted that this study has some limitations. Therefore, further research, including the evaluation of mercury content measurement, is needed to provide a more comprehensive understanding of the potential role and mechanism of action of CHR on testicular damage. Although CHR showed protective effects against HgCl_2_-induced testicular damage, the parameters NF-κB, TNF-α, IL-1β, Bax, Caspase-3, Bcl-2, ATF-6, and GRP78 were not evaluated at the protein level. Therefore, further research involving the evaluation of protein levels of NF-κB, TNF-α, IL-1β, Bax, Caspase-3, Bcl-2, ATF-6, and GRP78 parameters is needed to provide a more comprehensive understanding of Poly’s potential role and mechanism of action in testicular damage. Future studies should further validate these limitations by addressing them.

## Data Availability

Data will be made available on request.
